# P-315. Missed Opportunities for Pre-Exposure Prophylaxis Initiation in Persons with Newly Diagnosed HIV Infection at Henry Ford Health

**DOI:** 10.1093/ofid/ofaf695.534

**Published:** 2026-01-11

**Authors:** Kyle G Crooker, Sanjana Rao, Jamie Roberman, Brianna Hohmann, Smitha Gudipati, Indira Brar

**Affiliations:** Henry Ford Hospital, Detroit, MI; Wayne State University School of Medicine, Detroit, Michigan; Henry Ford Health/Michigan State University Health Sciences, Detroit, Michigan; Henry Ford Health, Detroit, Michigan; Henry Ford Health System, Detroit, MI; Henry Ford Hospital, Detroit, MI

## Abstract

**Background:**

HIV prevalence in Michigan continues to increase as the number of new diagnoses exceeds the number of deaths, with the highest rates occurring in Detroit. In Michigan, PrEP uptake is determined as low to medium, resulting in HIV diagnoses declining by 0.9% as compared to 8% in states with high PrEP uptake. HIV screening initiatives such as HIV testing in emergency departments (EDs) are effective in new case identification; however, opportunities for PrEP initiation are missed. The aim of this study was to identify missed opportunities for the use of PrEP in newly diagnosed persons with HIV (PWH).

**Table 1:**
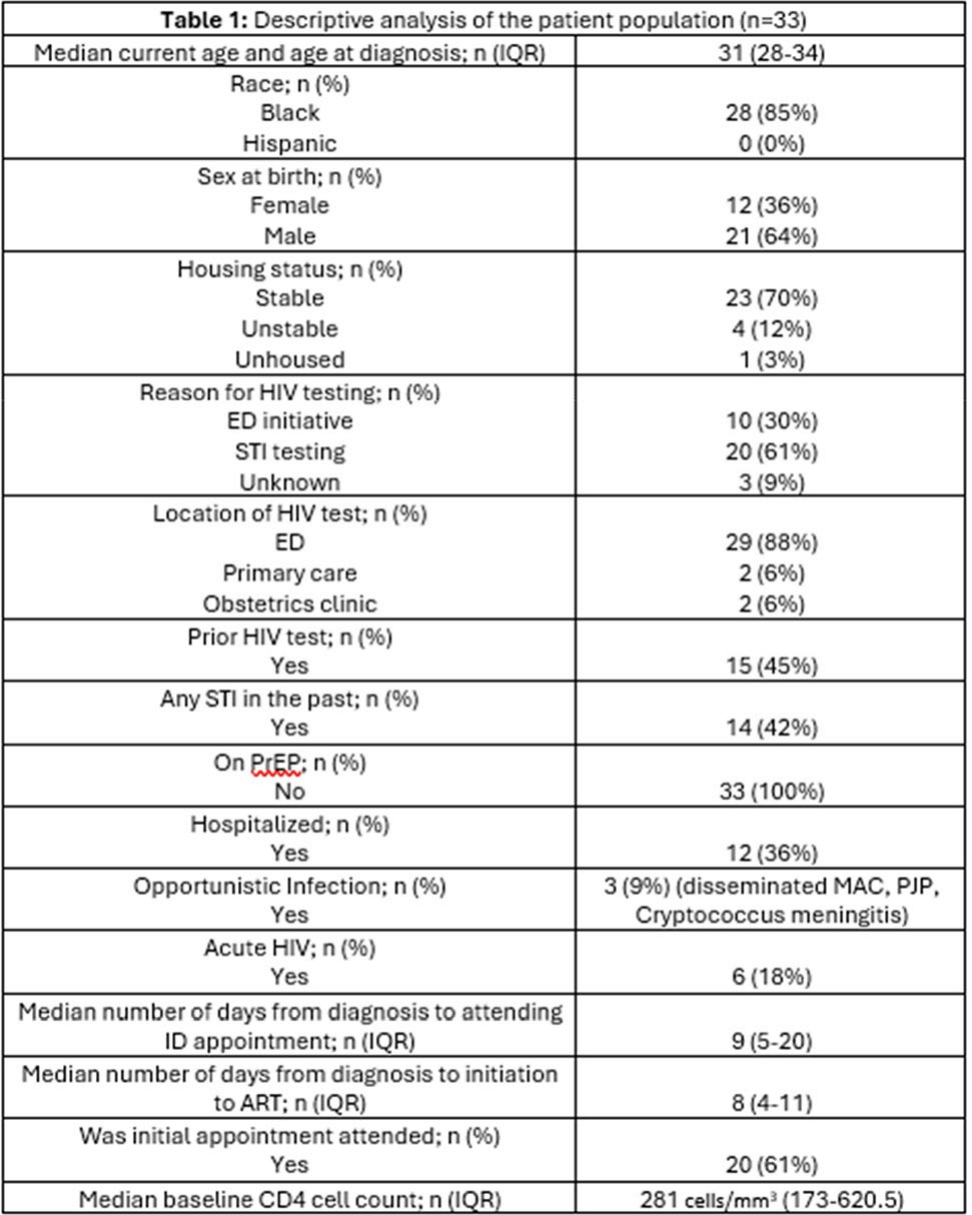
Descriptive analysis of the patient population

**Table 2:**
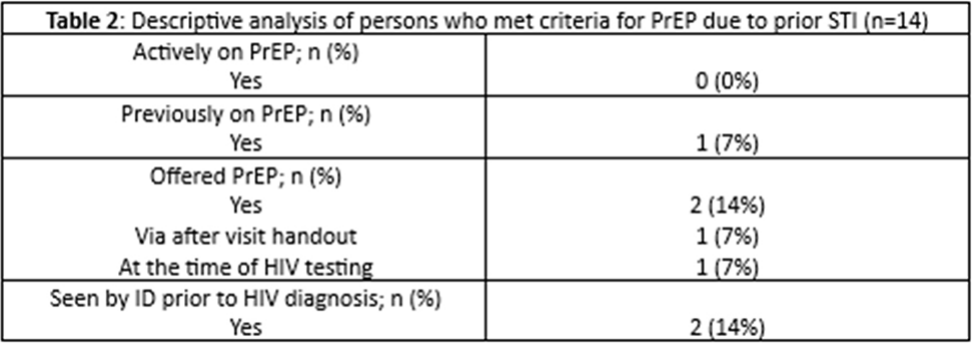
Descriptive analysis of persons who met criteria for PrEP due to prior STI

**Methods:**

This was a retrospective observational study of adults with newly diagnosed HIV at Henry Ford Health in Detroit, Michigan from 1/1/2024 to 8/1/2024. Testing indications in the ED included sexually transmitted infections (STI) or an ED screening initiative where a best practice alert prompted the providers to order a HIV screening test for individuals 18-65 years without a previous HIV test. Electronic health records were reviewed to collect demographic and clinical data.

**Results:**

Thirty-three individuals with a new HIV diagnosis were included. Demographics are seen in Table 1. The majority (88%) of diagnoses were made in the ED with the highest rates of incidence in Wayne County. Median baseline CD4 cell count was 281 cells/mm^3^; 9% of individuals had an opportunistic infection. Median time to first HIV appointment and ART initiation were 9 and 8 days in 66% of individuals. However, 34% individuals were lost to follow up. None of the individuals were actively on PrEP; 14 had a prior STI and only 2/14 were offered PrEP. Table 2 shows a descriptive analysis of individuals eligible for PrEP.

**Conclusion:**

While we have made progress in screening for HIV and linking newly diagnosed PWH to care, providing PrEP for HIV to prevent infection has not progressed as evidenced by our study. Despite multiple interactions with healthcare systems a significant majority had no discussion about PrEP. These missed opportunities underscore the need for increased efforts to provide high risk populations with tools including PrEP to reduce risk for HIV infection. To achieve this goal, innovative strategies to prevent new HIV infections are needed, including telePrEP and increased primary care outreach.

**Disclosures:**

Indira Brar, MD, Gilead: Advisor/Consultant|Gilead: Grant/Research Support|Gilead: Honoraria|ViiV Healthcare: Advisor/Consultant|ViiV Healthcare: Grant/Research Support|ViiV Healthcare: Honoraria

